# Worms and the Treatment of Inflammatory Bowel Disease: Are Molecules the Answer?

**DOI:** 10.1155/2008/567314

**Published:** 2008-05-21

**Authors:** Nathalie E. Ruyssers, Benedicte Y. De Winter, Joris G. De Man, Alex Loukas, Arnold G. Herman, Paul A. Pelckmans, Tom G. Moreels

**Affiliations:** ^1^Laboratory of Experimental Medicine and Pediatrics, Division of Gastroenterology, University of Antwerp, 2610 Antwerp, Belgium; ^2^Division of Infectious Diseases, Queensland Institute of Medical Research, Brisbane, QL 4029, Australia; ^3^Laboratory of Pharmacology, University of Antwerp, 2610 Antwerp, Belgium; ^4^Division of Gastroenterology and Hepatology, University Hospital of Antwerp, Wilrijkstraat 10, 2650 Edegem, Belgium

## Abstract

The lack of exposure to helminth infections, as a result of improved living standards and medical conditions, may have contributed to the increased incidence of IBD in the developed world. Epidemiological, experimental, and clinical data sustain the idea that helminths could provide protection against IBD. Studies investigating the underlying mechanisms by which helminths might induce such protection have revealed the importance of regulatory pathways, for example, regulatory T-cells. Further investigation on how helminths influence both innate and adaptive immune reactions will shed more light on the complex pathways used by helminths to regulate the hosts immune system. Although therapy with living helminths appears to be effective in several immunological diseases, the disadvantages of a treatment based on living parasites are explicit. Therefore, the identification and characterization of helminth-derived immunomodulatory molecules that contribute to the protective effect could lead to new therapeutic approaches in IBD and other immune diseases.

## 1. INFLAMMATORY BOWEL DISEASES AND THE HYGIENE HYPOTHESIS

Inflammatory bowel diseases (IBDs), such as Crohn's disease and ulcerative colitis, are chronic immune diseases of the gastrointestinal tract. Although the aetiologies of these diseases still remain unknown, the current hypothesis indicates that IBD results from an uncontrolled immune response to the normal gut flora [1, 2]. Genetic factors and environmental factors both contribute to the damaging mucosal immune response [3, 4].

The incidence of IBD has steadily increased in the developed world since 1950 [5, 6]. According to the *hygiene hypothesis*, this is directly related to the higher hygienic standards in these countries [7, 8]. It is suggested that the lack of exposure to infectious agents like helminths, as a result of improved living standards and medical conditions, modulates the development of the immune system and thereby increases the risk of immune diseases [9, 10].

The hygiene hypothesis was initially proposed by Strachan in 1989 for hay fever [11] and additional epidemiological studies were performed to further investigate the link between this hygiene concept and the incidence of other immunological diseases. As a consequence, the hygiene hypothesis is now proposed for several immunological disorders such as asthma and allergic diseases [12], cardiovascular diseases [13], Type 1 diabetes mellitus [14], multiple sclerosis [15], and IBD [16].

The hygiene hypothesis for IBD is clearly supported by the geographical distribution of the disease. There is a well described north-south gradient for the incidence of IBD. Northern Europe and North America have the highest IBD incidence rates whereas Crohn's disease and ulcerative colitis remain scarce in South America, Africa, and Asia [6, 17]. However, the gap between high- and low-incidence areas in northern versus southern regions is narrowing. In Asia, for example, incidence rates still remain low as compared to Europe, but they are rapidly increasing [18]. Changing lifestyle is thought to be the major cause of the disease increase in low-incidence areas [18]. The most important factor to explain these geographical differences is the socioeconomic level [16]. IBD is more frequently seen among patients with a higher socioeconomic status [19, 20]. Higher socioeconomic levels can be associated with better sanitation conditions, high-quality water, and better medical standards [2].

Another factor supporting the hygiene hypothesis is the inverse relationship between infant mortality rates and the incidence of IBD. Infant mortality might be linked to worse hygiene and medical conditions. Countries with high infant mortality rates consequently have lower reported incidence of IBD [21].

As mentioned previously, better hygienic circumstances translate into diminished exposure to infectious agents like helminths. The absence of such parasitic infections during childhood renders the immune system more prone to allergic and immune diseases. Thus infections seem to activate an important protective factor against these disorders [7]. Identifying the nature of this protective effect and implementing this notion in therapeutic strategies against IBD and other immune diseases is now the challenge for basic research.

## 2. IMMUNOLOGY OF THE GASTROINTESTINAL TRACT

### 2.1. Initiating innate and adaptive immune responses to enteric antigens in the gut

The gastrointestinal tract is continuously exposed to a wide range of dietary and environmental antigens, both harmless and pathogenic. Mounting protective immune responses against harmful pathogens whilst also preventing excessive responses to harmless antigens from food and bacterial flora is one of the major dichotomous functions of the mucosal immune system [22].

There are different levels of host defence that pathogens have to trespass to induce inflammation. Numerous mechanisms are acting to form a physical barrier to prevent micro-organisms from gaining access to the underlying tissues. Production of saliva and mucus, gastric and pancreatic juices, intestinal peristalsis, and epithelial cells all contribute to the elimination of pathogens from the gut lumen [23, 24]. Tight junctions between epithelial cells form a barrier to prevent bacterial pathogens from invading the gut tissue [25–27]. Once a pathogen breaks through this physical barrier, innate and adaptive immune responses work closely together to eliminate the intruder [28].

Antigens in the gut lumen can be taken up via different transport routes [29]. The innate immune system will respond to pathogen associated molecular patterns (PAMPs). As a part of the innate immune system, phagocytes like monocytes, macrophages and dendritic cells, and cytotoxic cells like natural killer cells rapidly control the invasion [30]. The adaptive immune system responds to antigens which have been presented by cells of the innate immune system [30]. Once antigens are taken up by antigen presenting cells, such as dendritic cells, fragments of the antigen are presented to T-cells locally or in mesenteric lymph nodes (MLNs) after migration of the antigen presenting cells [31, 32]. Adaptive immune responses are initiated by stimulation of lymphocytes. T-cells will help B lymphocytes to secrete immunoglobulins, the antigen-specific antibodies that are responsible for eliminating extracellular pathogens. On the other hand, T lymphocytes eradicate intracellular pathogens and mediate, for example, antihelminth and allergic responses [23]. Adaptive immune responses improve on repeated exposure to a given antigen by the formation of B and T memory cells [28].

### 2.2. T cell subsets and the immunological basis of helminth therapy in IBD

**Figure 1:**
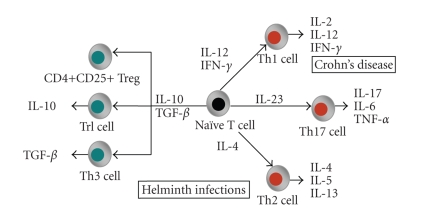
*T-cell subsets*. Naïve CD4+ T cells are stimulated by antigen presenting cells and the cytokine environment to proliferate into a certain subset. There are three distinct effector T-cell subsets (red): Th1, Th2, and Th17. CD4+ regulatory T-cells (green) can be subdivided in CD4+CD25+ Treg, Tr1, and Th3 cells. Crohn's disease is characterized by Th1, Th17 inflammation, whereas helminths induce Th2 and regulatory T-cells (modified from [36, 37]).

T lymphocytes are characterized by their cell-surface antigens called CD (cluster of differentiation) antigens. A common CD antigen found on all T-cells is the CD3 molecule which forms an essential part of the T-cell receptor and is important in the recognition of antigens presented by antigen presenting cells [33]. Within this pool of T lymphocytes, a difference is made between cytotoxic T-cells (CD8+) and helper T-cells (CD4+). CD4+ T-cells can orchestrate the functional activity of both innate and adaptive immune systems by “helping” macrophages, NK cells, CD8+ T cells, and B cells. These T helper (Th) cells can be divided into several subsets of CD4+ cells and each subset is suited for coordinating the effector activities that best combat the invading pathogen [34]. CD4+ T lymphocytes can be classified into distinct populations based on the cytokines they produce (Figure 1) [35–37].

Effector CD4+ T cells are divided into three distinct lineages. T helper 1 (Th1) cells are engaged in the eradication of intracellular pathogens (e.g., intracellular bacteria and viruses) and are characterized by the production of IL-2, IL-12, and IFN-*γ* [38]. Gastrointestinal inflammation during Crohn's disease is Th1 mediated [23]. T helper 2 (Th2) cells stimulate B-cell antibody production, eosinophil recruitment and mucosal expulsion mechanisms and are characterized by the secretion of IL-4, IL-5, and IL-13 [38]. Th2 cells enhance elimination of parasitic helminth infections and support allergic responses. During helminth infection, the host evokes a strong Th2 immune response to provide protection against worm colonization [39]. The cytokines produced by Th1 and Th2 cells crossregulate each other's development and activity, for example, IFN-*γ* produced by Th1 cells amplifies Th1 development and inhibits proliferation of Th2 cells [35]. In this way, helminths can evoke an immune response that might be able to attenuate the Th1 response found during Crohn's disease.

A third lineage of effector CD4+ cells has been recently discovered and is characterized by the production of IL-17, the Th17 cell. IL-17 induces expression of many innate inflammatory mediators such as IL-6, acute phase proteins, granulocyte-colony stimulating factor, and prostaglandin E2. Th1 and Th2 cytokines can inhibit Th17 development, while Th1 and Th2 effector cells seem resistant to IL17 expression [40]. It is now clear that the Th17 pathway is critical for the development of inflammation. IL17 is elevated in a variety of inflammatory conditions as shown for rheumatoid arthritis, asthma, and recently IBD [41]. Furthermore, it has been shown that IL-23 supports the proliferation of Th17 cells. IL-23 is mainly produced by activated myeloid cells such as macrophages and dendritic cells. The discovery of this new IL-23/IL-17 pathway was a major breakthrough in the immunopathogenesis of IBD and the exact role of this axis needs to be further defined [41, 42]. Investigation of the effect of helminth infections on the IL-23/IL-17 pathway may uncover additional immunological pathways by which helminths can provide protection against immune disorders.

Aside from these effector T-cells, another population of CD3+ cells called regulatory T (Treg) cells have been described. Treg cells have immunosuppressive function and cytokine profiles distinct from either Th1, Th2, or Th17 T-cells [43]. By suppressing excessive Th1, Th2, or Th17 immune responses, Treg cells play an important role in the maintenance of self-tolerance, thus preventing autoimmune diseases, as well as inhibiting harmful inflammatory diseases such as asthma and inflammatory bowel disease [44]. There is emerging evidence that distinct subgroups of CD4+, CD8+, and natural killer T cells mediate immune regulatory mechanisms [45]. The most attention is being paid to the CD4+ Treg cells which can be subdivided into different subsets. These include the natural CD4+CD25+ Treg cells, which inhibit immune responses through cell-cell contact and through the production of immunosuppressive cytokines, type 1 Tr (Tr1) cells which secrete high levels of IL-10 and type 3 T (Th3) cells which primarily secrete TGF-*β* [43]. Treg lymphocytes suppress the differentiation of both Th1 and Th2 lymphocytes and are considered real gatekeepers of the mucosal immune response [2].

The balance between Th1, Th2, and Treg cells is of special interest in the gastrointestinal immune system. The gut provides a unique microenvironment prone to Treg cell differentiation. This microenvironment is characterized by the constant exposure to commensal flora and food antigens and by the presence of immunomodulatory factors and cytokines that participate in the differentiation of the mucosal immune system [22]. Defects of the regulatory mechanisms may lead to development of specific Th1- or Th2- mediated diseases.

Given that helminths induce a distinct immunological mechanism compared to IBD, worms can be used as immunomodulators to downregulate the immune response in IBD. Helminths induce Th2 and Treg cells which are capable of suppressing Th1 effector cells, the cells responsible for maintenance of inflammation in IBD patients.

## 3. HELMINTHS AS THERAPEUTIC AGENTS IN IBD

### 3.1. Experimental and clinical studies supporting helminth-based therapy

Helminths colonize more than one third of the world population [46]. In developed countries, these parasites have been largely eradicated as a public health concern due to the availability of efficacious drugs and better sanitation conditions [47]. In developing countries, however, helminth colonization is still common [48]. As shown by epidemiological studies, there is an inverse relation between the frequency of worm colonization and the prevalence of IBD [46]. It was Elliott et al. who first proposed the hypothesis that the loss of exposure to parasitic worms increased the risk of IBD [49, 50].

Preliminary data of Elliott et al. illustrating a protective response of *Schistosoma mansoni* infection on trinitrobenzene sulphate (TNBS)-induced colitis in mice [49] have led to several experimental animal studies investigating the effect of helminth infections on IBD [2]. The first full study on helminth modulation of experimentally induced colitis was published by Reardon et al. in 2001. They showed that infection of mice with the tapeworm *Hymenolepis diminuta* ameliorated dextran sodium sulphate (DSS)-induced colitis [51]. Khan et al. subsequently showed that infection with the nematode, *Trichinella spiralis*, protected mice from colitis induced by intrarectal challenge with dinitrobenzene sulphate (DNBS) [52]. Elliott et al. demonstrated that schistosome eggs had a protective effect on TNBS-induced colitis in mice [53] and that *Heligmosomoides polygyrus* could reduce established colitis in IL-10 deficient mice [54]. We previously demonstrated a protective effect of infection with the blood fluke, *Schistosoma mansoni*, on trinitrobenzene sulfonic acid (TNBS)-induced colitis in rats [55]. Taken together, different helminth parasites (nematode, cestode, and trematode) can ameliorate colitis in different experimental animal models [56]. Furthermore, helminths also protect against other immunological diseases as shown in rodent models for asthma [57], type 1 diabetes mellitus [14], and experimental autoimmune encephalomyelitis [17, 58].

Based on the promising findings of helminth infections on experimental colitis, clinical studies were initiated. Treatment of patients with the porcine whipworm, *Trichuris suis*, resulted in clinical amelioration of both Crohn's disease and ulcerative colitis [59, 60]. In the same line, a proof of concept study showed clinical efficacy of experimental infection with the human hookworm *Necator americanus* on Crohn's disease [61]. Clinical trials of *Necator americanus* in asthma are being organized [62]. An international multicentre clinical trial is in preparation (awaiting FDA approval) to further investigate the clinical efficacy of helminth-based therapy in IBD [2].

### 3.2. The use of helminth-derived molecules as therapeutic agents

Although helminth infections appear to be effective against IBD, treatment of patients with living helminths may envision drawbacks. Persistent infection and/or invasion of the parasite (particularly zoonotic ones) to other tissues in the human host, where they might cause pathology, should be considered [63, 64]. In 2006, Kradin et al. reported that treatment of a pediatric Crohn's disease patient with five oral doses of *Trichuris suis* ova caused infection with living sexually immature worms in the ileocecal region and a sexually mature male worm within the cecum [64]. Although helminths may be beneficial in the treatment of IBD, using living helminth ova can lead to infection, therefore, therapeutic human helminth colonization needs to be closely examined for potential adverse side effects. Furthermore, intestinal helminths influence gastrointestinal physiology. Infection with certain nematodes may induce enhanced intestinal propulsive activity, goblet cell hyperplasia, and increased mucus secretion [65]. As a consequence, intestinal helminths may alter gastrointestinal motility, possibly resulting in intestinal symptoms like diarrhoea and abdominal cramps [65]. Moreover, the idea of being infected with a living parasite could be psychologically hard to accept for some patients. Therefore, treatment with immunologically active helminth molecules might overcome the possible disadvantages of a therapy with living parasites.

Identification and characterization of helminth-derived immunomodulatory molecules that contribute to the anticolitis effect could lead to new therapeutic approaches in IBD without the need for helminth infection [56, 66]. Using parasite extracts or synthetic drugs designed to mimic the disease-modulating effect of helminth molecules also allows greater flexibility in dosing routes and therapeutic applications [67].

Helminths possess evolved mechanisms to turn off proinflammatory cascades by secreting and expressing certain molecules [37]. Multiple studies have characterized a broad spectrum of helminth-derived immunomodulatory products. A detailed review of these products is beyond the scope of this paper so we will bring only some molecules of interest into focus. Maizels et al. showed that the filarial nematode *Brugia malayi* produces homologues of the mammalian cytokine TGF-*β*. Bm-tgh-2 is secreted by adult worms and binds to mammalian TGF-*β* receptors thus performing an immunomodulatory function in the host [39]. Helminths secrete cysteine protease inhibitors which interfere with antigen presentation and increase IL-10 secretion from macrophages [68]. Helminth-derived carbohydrates contribute to the induction of Th2 immune responses [69]. Lacto-N-fucopentaose III is the predominant carbohydrate component of *Schistosma mansoni* egg antigens and this glycan stimulates the secretion of Th2 cytokines [70]. Harnett et al. recently showed that the phosphorylcholine part of the glycoconjugate ES-62, secreted by filarial nematodes, is responsible for its anti-inflammatory action in arthritis [71]. Research focusing on the development of vaccines against helminth infections also showed the effectiveness of helminth antigens as immunomodulators. Vaccination studies against Schistosomiasis are focusing on the protective effect of several *Schistosoma* antigens [72]. Vaccination studies against human hookworm infections tested recombinant excretory/secretory (ES) products from L3 larval stages of *Ancylostoma caninum* and promising results were observed [73–75]. Furthermore, vaccination studies against hookworm infection revealed that administration of a cocktail of recombinant antigens has an improved protective effect compared to the protection achieved with separate antigens [76].

**Figure 2:**
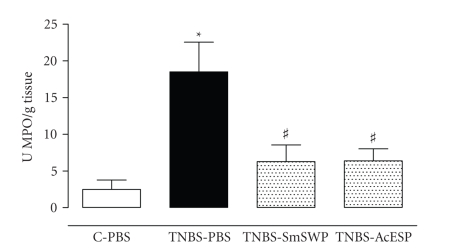
*Effect of Schistosoma mansoni soluble worm proteins (SmSWPs) and Ancylostoma caninum excretory/secretory products (AcESPs) on myeloperoxidase (MPO) activity*. MPO activity was measured to monitor the degree of myeloid cell infiltration in the colon. Data are presented as units MPO per gram of colon tissue and 1 unit equals the amount of MPO necessary to degrade 1 *μ*mol of H_2_O_2_ to H_2_O per minute at 25°C. TNBS-induced colitis caused a significant increase in MPO activity compared to control mice treated with phosphate-buffered saline (PBS). Intraperitoneal injection of helminth-derived products significantly ameliorated inflammation as shown by the significant decrease in MPO activity. Treatment of control mice with SmSWP or AcESP had no effect (data not shown). ∗ *p* ≤ .05, significantly different from contol PBS; # *p* ≤ .05, significantly different from TNBS-PBS; two way ANOVA, *n* = 7 − 10 [77].

In respect to IBD, there is need for in-depth experimental studies on the effect of helminth antigens on colitis. We are currently investigating the therapeutic potential of protein mixtures of *Schistosoma mansoni* and *Ancylostoma caninum* on TNBS-induced colitis in mice. As shown in Figure 2, preliminary experiments showed that both *S. mansoni* soluble worm proteins and *A. caninum* ES products attenuated TNBS-induced inflammation of the murine colon [77]. These results indicate that the beneficial effect of treatment with living worms on experimental colitis may be reproduced with soluble extracts of helminths. Yang et al. showed that *Schistosoma japonicum* egg antigens inhibited the development of asthma in a murine model [57]. *S. mansoni* antigens are also able to modulate innate immune responses and prevent onset of type 1 diabetes [78]. These studies indicate that treatment with helminth extracts may be as effective as treatment with living helminths and that the achieved protection is not specific for just one helminth species. Isolated helminth proteins may provide a more readily acceptable form of therapy for patients than living worms.

## 4. CONCLUDING REMARKS

The hygiene hypothesis suggests an inverse relationship between parasitic infections and the incidence of IBD. Epidemiological, experimental, and clinical data sustain the idea that helminths could provide protection against IBD. The importance of regulatory pathways such as regulatory T-cells, by which helminths induce such protection have been described. However, the complex pathways helminths activate to regulate the host's immune system need further investigation. Helminths influence innate as well as adaptive immune responses and this knowledge can contribute to new therapeutic approaches of helminth-induced protection. Therapy with living helminths appears to be effective in several immunological diseases. A logical next step, to avoid the possible disadvantages of a treatment with living parasites, is the identification and characterization of helminth-derived immunosuppressive molecules that contribute to the protective effect.
